# The beliefs of Senegal’s physicians toward the use of telemedicine

**DOI:** 10.11604/pamj.2019.34.97.20216

**Published:** 2019-10-16

**Authors:** Birama Apho Ly, Ronald Labonté, Ivy Lynn Bourgeault

**Affiliations:** 1 Faculty of Pharmacy, University of Sciences, Techniques and Technologies of Bamako, Bamako, Mali; 2 Faculty of Medicine, University of Ottawa, Ontario, Canada; 3 Telfer School of Management, University of Ottawa, Ontario, Canada

**Keywords:** Beliefs, physicians, telemedicine, theory of planned behaviour, Senegal, Africa, district health centre, hospital

## Abstract

**Introduction:**

Telemedicine is seen as a potential solution to improve access to specialist services in underserved areas, but using telemedicine depends on physicians' beliefs regarding its use. Applying the theory of planned behaviour, there are three kinds of beliefs of relevance: behavioural, normative and control beliefs. This study aimed to determine the behavioural, normative and control beliefs of Senegal's physicians regarding the use of telemedicine.

**Methods:**

A qualitative descriptive study involving individual interviews with physicians was conducted between January and June 2014. It included 32 physicians working in public hospitals and 37 physicians working in district health centres. Interviews were taped, transcribed and their content coded thematically using the NVivo 10 software.

**Results:**

The most significant positive behavioural belief was that telemedicine makes experts' opinions accessible despite distance; the most important negative behavioural belief was that telemedicine can lead to medical errors. The positive normative belief mentioned most was that patients approve the use of telemedicine, but the negative normative belief mentioned most was that the patients would not approve it. The prevailing positive control belief was that physicians will use telemedicine if it is easy to use and the most cited negative control belief was that physicians will not use telemedicine if they have insufficient time.

**Conclusion:**

The results of this study provide a better understanding of the beliefs of Senegal's physicians regarding telemedicine, which can help in designing interventions to promote its use. Such interventions may help improve access to healthcare in rural areas.

## Introduction

Physicians are unevenly distributed in Senegal; only 24% of them working in rural areas where 60% of the country's population lives [1]. This uneven distribution leads to unequal access to healthcare and consequently to health inequalities [2]. One of the leading causes of this uneven distribution of physicians is the failure to both recruit and retain them in rural areas. This failure is influenced by many factors; the best-known are contextual, economic, educational, family, individual, organizational and professional factors [3,4]. International migration also plays an important role with more than 51% of Senegalese-trained physicians work abroad [5]. During the last few decades, Senegal has adopted production and management measures to recruit and retain physicians in rural areas. Production measures include the opening of private medical schools and the provision of fellowships to students who accept to practice in rural areas [3]. Management measures include the creation of the Directorate of Human Resources (DHR) in 2004 within the Ministry of Health to improve the coordination of human health resources. It also includes the revision of the status of public health workers by a commission comprised of the representatives of the Ministry of Health, the Ministry of Civil Service and union activists in order to improve the professional and educational conditions of these workers. Finally, there has been a provision of financial incentives and transportation subsidies to rural physicians [3]. To date, none of these measures has resulted in a more equitable distribution of physicians between rural and urban areas. In this context, telemedicine, the practice of medicine from a distance through information and communication technologies (ICT) [6], could be a useful means to recruit and retain physicians, and to improve access to healthcare in rural areas [4]. The potential success of telemedicine in this regard depends, in part, on physicians' beliefs regarding its use, on which little is known at present. The purpose of this study, then, was to determine Senegalese physicians' beliefs concerning the use of telemedicine drawing upon the theory of planned behavior (TPB) which distinguished between behavioural, normative and control forms. Behavioural beliefs refer to the subjective probability that behaviour will produce a given outcome.

Normative beliefs refer to the perceived behavioural expectations of important referents, including individuals or groups. Control beliefs refer to the perceived presence of factors that may facilitate or impede the performance of the behaviour [7]. This study focuses on physicians working in the public sector only, particularly those working in public hospitals and district health centres, as logistical limitations precluded inclusion of physicians working in the private sector. Existing research, suggests that with regard to behavioural beliefs, telemedicine is considered to reduce the transportation of patients, saving costs [8-10], while also facilitating access to continuing education, research and professional exchanges [9-11]. Telemedicine is seen as a means to improve the quality of health services and to reduce medical errors [9,11,12], but is also suspected to have a negative impact on data security, the physician/patient relationship and physician work overload [8,10,13]. Telemedicine is equally considered as potentially leading to medical errors, and as an inefficient means to guarantee a good income for physicians [14]. In relation to the normative beliefs, some studies suggest that nurses, physicians and patients are all likely to approve of telemedicine use, [15-17], although others believe that both physicians and patients may be opposed to its use [17]. With respect to control beliefs, a determining factor may be the ease of equipment use, physicians' existing familiarity with computers, and their access to handheld devices (e.g. a smartphone) and the internet [15,18]. Data security, data conservation, technical problems, a lack of training, and patient opposition to telemedicine services are all thought to negatively affect perceived control [13,19-21]. Most of these studies were carried out in high-income countries. Little is known about the transferability of their findings to low-income countries such as Senegal. This article reports on the first such study exploring such beliefs amongst practicing physicians in Senegal, with potentially transferable findings to other African countries at a similar level of health system development.

## Methods

**Study design, participants, and sampling:** to identify Senegalese physicians' beliefs about telemedicine use, we conducted a qualitative descriptive study involving individual interviews. Participants comprised physicians who practicing in public hospitals (total N = 596) and district health centres (total N = 187), based on 2013 data. Using purposive random sampling, 40 physicians were selected from those practicing in public hospitals and 40 from those practicing in district health centres. These physicians were contacted to establish their availability, willingness to participate in the study, and consent to be interviewed and audio-recorded. Thirdy-two physicians practicing in public hospitals and 37 practicing in district health centres accepted to participate in the study.

**Data collection:** participants were interviewed individually in person. All interviews were audio-recorded and took place in public hospitals, district health centres, physicians' homes, hotels, restaurants, the airport, training centres and at Cheikh Anta Diop University, depending on each physician's availability and preference. An interview guide was designed and used to facilitate the conduct of individual interviews. Physicians were asked about their positive (advantages of using telemedicine) and negative (inconvenience of using telemedicine) behavioural beliefs; positive (people who can approve their use of telemedicine) and negative (people who can disapprove their use of telemedicine) normative beliefs; and positive (facilitators of the use of telemedicine) and negative (barriers to the use of telemedicine) control beliefs. Table 1 outlines the different questions posed. During interviews, the principle was to allow the physicians to talk at length, in their own terms, and with enough time to reflect. Clarifications were sought whenever needed. Interviews lasted from 10 to 45 minutes and to interview physicians, authorization was secured from the Ministry of Health of Senegal (No 111 MSAS/ DPRS/ DR), the Research Ethics Board of the University of Ottawa (No H 09-13-12) and Senegal's National Ethics Committee on Health Research (No 205 MSAS/ DPRS/CNERS). Each physician provided written informed consent before answering our questions and their privacy and anonymity were respected during all the stages of the research.

**Table 1:** Interview guide for the study of physicians’ beliefs

No	Beliefs	Questions
Behavioral beliefs regarding the use of telemedicine		
1	Positive behavioral beliefs	What do you believe are the advantages of using telemedicine in your professional activities?
2	Negative behavioral beliefs	What do you believe are the disadvantages of using telemedicine in your professional activities?
**Normative beliefs regarding the use of telemedicine**		
3	Positive normative beliefs (positive normative referents)	Are there any individuals or groups of individuals who would approve of your decision to use telemedicine in your professional activities?
4	Negative normative beliefs (negative normative referents)	Are there any individuals or groups of individuals who would disapprove of your decision to use telemedicine in your professional activities?
**Control beliefs regarding the use of telemedicine**		
5	Positive control beliefs	What factors or circumstances would enable you to use telemedicine in your professional activities?
6	Negative control beliefs	What factors or circumstances would make the use of telemedicine in your professional activities difficult or impossible?

**Figure 1:**
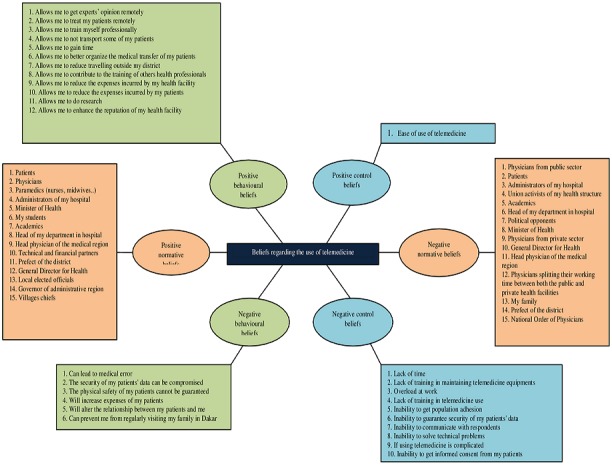
Thematic chart with the global, organizing and basic themes related to the beliefs of Senegal physicians

**Analysis:** data were analysed using the Attride-Stirling thematic network analysis framework [22]. This framework is a method of conducting a thematic analysis of qualitative or textual data which helps in identifying emergent concepts, themes, and relationships. This analysis involved several steps. First, interviews were transcribed by four assistants. Second, all transcripts were imported into NVivo 10 software, where data were both deductively and inductively coded by the lead author. Data coding continued until theoretical saturation was reached, that is, until no new concepts emerged. Third, the completed code structure was applied to develop broader conceptual themes. Finally, all the themes identified were collated into a thematic chart to reflect global, organizing and basic themes in line with the Attride-Stirling's thematic network analysis framework [22]. Figure 1 shows the thematic chart with the global (beliefs regarding the use of telemedicine), organizing (positive/negative behavioural, normative and control beliefs) and basic themes. Global and organizing themes were identified a priori based on the conceptual framework while basic themes were identified inductively. Physicians were then compared based on the type of their health facility (public hospitals and district health centres).

**Funding:** this research was finance by the Faculty of Graduate and Postdoctoral Studies (FGPS) of the University of Ottawa; Canadian Institutes of Health Research (grant number 106493); Telfer School of Management Research Funds (SMRF); and Global Health Practicum Grant (GHP).

**Ethical approval:** this study was approved by the Research Ethics Board of the University of Ottawa (No H 09-13-12). The protocol was approved by Senegal's National Ethics Committee on Health Research (No 205 MSAS/DPRS/CNERS).

## Results

**Socio-demographic and professional characteristics of participants:** Table 2 shows the socio-demographic and professional characteristics of the physicians. The 32 physicians working in public hospitals were selected from 13 public hospitals spread across five administrative regions. Their average age was 44, with a range of 29 to 61. They were all specialist physicians (32) with more male (25) than female (7) participants. The 37 physicians working in district health centres were selected from 37 district health centres spread across 12 administrative regions. Their average age was 40, with a range of 33 to 53. They were more specialist physicians (24) than general practitioners (13) again with more male (35) than female (2) physicians.

**Table 2:** Socio-demographic and professional characteristics of the physicians working in public hospitals and district health centers

Characteristics		Physicians working in public hospitals		Physicians working in district health centers	
		N	%	N	%
**Sex**	**Male**	25	78.13	35	94.59
	Female	7	21.87	2	5.40
**Age**	≤ 30	2	6.25	0	0
	31-35	3	9.37	10	27.03
	36-40	5	15.62	13	35.13
	41-45	7	21.87	7	18.92
	46-50	6	18.75	6	16.22
	51-55	8	25.00	1	2.70
	56-60	0	0.00	0	0.00
	> 60	1	3.12	0	0.00
**Specialization**	General practitioner	0	0.00	13	35.13
	Specialist physician	32	100	24	64.86
**Medical Region**	Dakar	26	81.25	2	5.40
	Outside Dakar	6	18.75	35	94.60

**N**: number, **%**: percentage

### Physicians' behavioural beliefs

**Positive behavioural beliefs:** Table 3 provides an overview of the positive behavioural beliefs and their number of mention by each group of physicians (those working in district health centres and those working in public hospitals). Physicians working in public hospitals believe that telemedicine allows them to access experts' opinions remotely, to treat their patients remotely, to train themselves continuously, to do research, to reduce their travel outside their work locations, and to save time. They also believe that telemedicine enables their patients' to avoid transportation costs and time and to better organize their patients' transfer if needed. They equally believe that telemedicine enables their health facility to save costs. Finally, they think that telemedicine can contribute to the training of other health professionals. In relation to remote treatment and exchanges with experts, one of the physicians working in public hospitals said: *"Well! with telemedicine, I can provide care to patients. I can give advice. I can share my impressions on radiographic images. I can propose a strategy to treat each patient. I can do all these things"* (male specialist physician working in a public hospital in Dakar). The physicians working in district health centres identified similar beliefs, for example: *"As I said, (telemedicine) benefits include knowledge exchanges between health professionals and saving patients' time and money"* (male general practitioner working in a district health centre outside Dakar). The only difference between hospital and district health centre responses was that in the latter locale physicians did not mention telemedicine's contribution to the training of other health professionals. Conversely, physicians in district health centres uniquely reported that the installation of telemedicine equipment (telecommunications and medicine equipment), which is essential to its use, will improve district health centre's the technical platform and hence its reputation amongst patients.

**Table 3:** Physicians’ behavioral beliefs

No	Beliefs	Physicians working in public hospitals	Physicians working in district health centers
**Positive behavioral beliefs**			
1	Allows me to get experts’ opinion remotely	23	21
2	Allows me to treat my patients remotely	22	30
3	Allows me to train myself professionally	19	29
4	Allows me to not transport some of my patients	14	17
5	Allows me to gain time	10	4
6	Allows me to better organize the medical transfer of my patients	9	7
7	Allows me to reduce travelling outside my district	8	7
8	Allows me to contribute to the training of others health professionals	7	0
9	Allows me to reduce the expenses incurred by my health facility	7	6
10	Allows me to reduce the expenses incurred by my patients	5	18
11	Allows me to do research	4	2
12	Allows me to enhance the reputation of my health facility	0	3
**Negative behavioral beliefs**			
1	Will mislead medically	9	8
2	The security of my patients' data can be compromised	3	3
3	The physical safety of my patients cannot be guaranteed	2	0
4	Will increase expenses of my patients	1	1
5	Will alter the relationship between my patients and I	0	4
6	Can prevent me from regularly visiting my family in Dakar	0	1

**Negative behavioural beliefs:** Table 3 provides an overview of the negative behavioural beliefs and their number of mention by group of physicians. Specialist physicians working in public hospitals express concern over telemedicine potential to lead to medical errors, data security problem, additional costs to patients and patients' physical safety risks due to the likelihood of increased medical tests generated through telemedicine use. One physician working in a public hospital, reflecting a frequently cited concern, commented that: *"The main drawback (with telemedicine) that I find is disclosure of patients' confidential medical information through communication technologies which are not totally secure"* (female specialist physician working in a public hospital in Dakar). In relation to patients' physical safety, another physician working in a public hospital said: *"Patients need to be protected. I mean that the prescription of various medical tests needs to be justified. People (physicians) will ask for medical tests that are not always needed and that can put patients in more danger. I think that this is part of telemedicine's inconvenience"* (male specialist physician working in a public hospital in Dakar). Physicians working in district health centres held the same beliefs except the one about the physical safety of their patients. Conversely, physicians working in district health centres uniquely commented on how telemedicine might affect their relationship with their patients by reducing patient trust in their abilities: *"Some patients think that we are supermen. They won't understand that we consult another physician (using telemedicine) to treat them. They could say that we are not skilled enough. They can become distrustful of us"* (male general practitioner working in a district health centre outside Dakar). Another concern for physicians working in district health centres was how telemedicine might prevent them from regularly visiting their families in Dakar by reducing their need to travel to Dakar for training, conference, or other practice-related reasons: *"telemedicine will reduce my moves outside my area, but my family, which is in Dakar, may dislike that. My wife and my children would like that I frequently come home because I don't live with them. Here, I live alone."* (male specialist physician working in a district health centre outside Dakar).

### Physicians' normative beliefs

**Positive normative beliefs:** Table 4 provides an overview of positive normative beliefs and their number of mention by group of physicians. Specialist physicians working in public hospitals generally believe that the people they engage with professionally are likely to approve of their telemedicine use: *"I believe that professionals who work in hospital, whether they are nurses, midwives, physicians or administrators, will approve telemedicine if procedures are well respected"* (male specialist physician working in a public hospital in Dakar). Those working in district health centres identified the same set of referents, but also mentioned their technical and financial partners, their district Prefect and village chiefs, their local elected officials, and even the Governor of their region as likely to approve of their telemedicine use, indicating a larger and more local network of normative referents.

**Table 4:** Physicians’ normative beliefs

No	Beliefs	Physicians working in public hospitals	Physicians working in district health centers
**Positive normative beliefs**			
1	Patients	16	20
2	Physicians	13	20
3	Paramedics (nurses, midwives.)	10	10
4	Administrators of my hospital	9	0
5	Minister of Health	7	17
6	Students	5	0
7	Academics	4	8
8	Head of my department in hospital	3	0
9	Head physician of the medical region	0	8
10	Technical and financial partners	0	4
11	Prefect of the district	0	3
12	General Director for Health	0	3
13	Local elected officials	0	2
14	Governor of the administrative region	0	1
15	Villages chiefs	0	1
**Negative normative beliefs**			
1	Physicians from the public sector	5	9
2	Patients	3	6
3	Administrators of my hospital	3	0
4	Union activists of my health structure	2	1
5	Academics	1	1
6	Head of my department in hospital	1	0
7	Political opponents	1	0
8	Minister of Health	0	5
9	Physicians from private sector	0	4
10	General Director for Health	0	4
11	Head physician of the medical region	0	3
12	Physicians who transit between public and private sectors	0	1
13	My family	0	1
14	Prefect of the district	0	1
15	National Order of Physicians	0	1

**Negative normative beliefs:** Table 4 provides an overview of negative normative beliefs and their number of mention by group of physicians. Some public hospital physicians had an opposite perception, believing that several referents (including patients, colleagues, political opponents, and administrators) might oppose their telemedicine use: *"Yes, if the implementation of telemedicine is expensive, the administrators of the hospital can cause problems"* (female specialist physician working in a public hospital in Dakar). Union activists of their hospital (concerned over employment impacts) and medical academics might also raise objections. Physicians in district health centres identified similar referents apart from those associated directly with hospital administration. They also identified many of the same “positive” referents as potentially having a negative impact, including their private sector colleagues or those splitting time between both private and public, who may see telemedicine as introducing a new competitive element.

### Physicians' control beliefs

**Positive control beliefs:** only one positive control belief was identified: the ease of use of telemedicine which refers to how easily physicians can use telemedicine equipment (Table 5). *"If it is something easy in which I will not waste too much time, for example, to start the connection or to download something, I am ready to use it"* (male specialist physician working in a public hospital in Dakar).

**Table 5:** Physicians’ control beliefs

No	Beliefs	Physicians working in public hospitals	Physicians working in district health centers
**Positive control beliefs**			
1	Ease of use of telemedicine	2	5
**Negative control beliefs**			
1	Lack of time	7	9
2	Lack of training in maintaining telemedicine equipments	5	6
3	Overload at work	4	8
4	Lack of training in telemedicine use	4	7
5	Inability to guarantee security of my patients' data	4	6
6	Inability to communicate with respondents	3	12
7	Inability to solve technical problems	3	6
8	If using telemedicine is complicated	2	1
9	Inability to get informed consent from my patients	1	6

**Negative control beliefs:** Table 5 provides an overview of negative control beliefs and their number of mention by group of physicians. Several negative control beliefs were mentioned. Physicians working in public hospitals believe that a lack of time, of training in telemedicine use and equipment maintenance, and work overload could prevent them from using it. Regarding the lack of time, one physician said: *"You know, we are in particular conditions of medical practice. We handle a large volume of patients. So, time is not enough. We are, all the time, busy with patients, consultations or other things. I think we do not have enough time, which can prevent us from using telemedicine"* (male specialist physician working in a public hospital outside Dakar). These physicians also said that they would not use telemedicine, or will stop using it, if patient data security is not guaranteed, if they are unable to communicate with respondents via telemedicine at the time they need them, if they are not able to solve technical problems (internet, electricity and equipment), or if patients do not give their consent for the use of telemedicine. Physicians working in district health centres expressed the same concerns.

## Discussion

Our results show that Senegal's physicians perceived both advantages (positive behavioural beliefs) and disadvantages (negative behavioural beliefs) of using telemedicine in their professional activities. The results are broadly consistent with those of the literature reviewed earlier, although that advantages and disadvantages were often contradictory (e.g. telemedicine has the potential to reduce medical error while also increasing medical error); this could indicate some continuing discomfort or uncertainty about telemedicine, at least across the physician population as a whole. Further probing of these apparent contradictions would be useful in any initiative to increase telemedicine use in the country. That physicians mentioned twice as many advantages as disadvantages suggests that Senegal's physicians may have a more positive than negative attitude toward telemedicine use. The theory of planned behaviour, however, posits that a positive intention also depends on normative and control beliefs. As normative beliefs, many different individuals and groups were identified, and were thought to be supportive but, again and somewhat paradoxically, many were recognized, at the same time and by the same physician being interviewed, as being both a supporter and an opponent. These results coincide with those of researchers that found that nurses [15], physicians [15-17] and patients [16,17] can approve the use of telemedicine, but that physicians and patients can also disapprove it [17]. This may reflect differences amongst our physician sample (some seeing the same referent group as a positive, others as a negative, influence) but it could also attest to physicians recognizing the variability that might exist within their referent group, or in the case of patients, specific individuals, or specific patient/physician encounters. Nonetheless, the breadth of referent groups identified infers that the development of telemedicine in Senegal will require a multi-sectoral approach involving quite a large number of individuals and groups, and awareness campaigns targeting referent groups on the necessity of their support for telemedicine use by physicians. Control beliefs refer to the factors that can encourage or prevent the use of telemedicine by physicians. The control belief regarding ease of use was considered as the only factor that would encourage telemedicine use by our participants. Several others beliefs related to complexity of the technology, training, equipment maintenance or technical difficulties were all seen as impediments to its use, consistent with findings from other studies. These results indicate that Senegal's physicians perceive more barriers than facilitators, which is likely to negatively affect their perceived behavioural control and hence use of telemedicine (Figure 1). According to the Theory of Planned Behaviour, perceived behavioural control can have a direct or indirect influence on individuals' behaviour [23], with other studies finding that perceived control can influence physicians' behaviour regarding telemedicine [24-26].

## Conclusion

This study showed that Senegal's physicians have several behavioural, normative and control beliefs regarding the use of telemedicine and demonstrated that the theory of planned behaviour can be usefully applies in an African context, in this particular study, that of Senegal's public hospitals and district health centres. In providing a better understanding of physicians' beliefs, study results can assist those planning future telemedicine strategies in Senegal with clearer guidance as to what may enable or present a barrier to physician use of the technology. To the extent that telemedicine development in the country accounts for physician beliefs such that it improves their uptake of the technology, this could improve population access to specialist services in historically underserved areas.

### What is known about this topic

- Regarding the difficulty to equitably distribute physicians between rural and urban areas, telemedicine is considered as a good means to improve access to physicians, particularly specialist physicians by acting positively on their recruitment and retention in rural areas;
- The potential success of the use of telemedicine depends on physicians' beliefs, particularly physicians' behavioural, normative and control beliefs toward its use, on which little is known at present;
- The literature provides some information on physicians' behavioural, normative and control beliefs toward the use of telemedicine.

### What this study adds

- Telemedicine was considered as a good tool to get experts' opinion and treat patients remotely, train himself professionally, gain time, better organize and reduce the number of medical transfers, reduce travelling outside its workplace, train other health professionals, reduce the expenses incurred by health facility and patients, do research and enhance the reputation of health facility;
- The head physician of medical regions, technical and financial partners, prefects of districts, general Director for Health, local elected officials, Governors of administrative regions and villages chiefs were considered as supporters of telemedicine in District Health Centres but not in public hospitals;
- The only identified facilitator was the ease of use of telemedicine.

## Competing interests

The authors declare no competing interests.
